# Geospatial Investigation of Nigerian Honey and Detection of Anti-Enteric Biomarker

**DOI:** 10.1155/2020/9817673

**Published:** 2020-04-28

**Authors:** P. Akinniyi Akinduti, Oluwaseun Ejilude, Joseph Olugbuyiro, Adeyemi G. Adewale, Okanlawon Onagbesan, Oluwadun Afolabi

**Affiliations:** ^1^Microbiology Unit, Department of Biological Sciences, Covenant University, Ota, Nigeria; ^**2**^ Microbiology Laboratory, Sacred Heart Hospital, Lantoro, Abeokuta, Nigeria; ^**3**^ Department of Chemistry, Covenant University, Ota, Nigeria; ^**4**^ Department of Civil Engineering, Covenant University, Ota, Nigeria; ^**5**^ Biotechnology Centre, Federal University of Agriculture, Abeokuta, Nigeria; ^**6**^ Department of Medical Microbiology and Parasitology, Olabisi Onabanjo University, Sagamu, Nigeria

## Abstract

Geospatial mapping and antibacterial biomarkers were investigated in Nigerian honey used for therapeutic purposes in several communities affected with prevalent antibiotic-resistant enteric bacilli. Randomly collected enteric bacilli from faecal samples were biotyped and phenotypically assayed for antibiotic resistance and profiled for R plasmids. R plasmid molecular weight and multiantibiotic resistance index (MARI) relatedness were evaluated for resistance among phylogroups. Honey cidal activity, time kill kinetics, and bioactive markers were determined and analysed for geospatial distribution. More than 30% enteric biotypes were resistant to cotrimoxazole, ciprofloxacin, and tetracycline at MIC ≥16 *μ*g/ml (*P*=0.004). Two unrelated cluster complexes with diverse antibiotic resistance indices expressed high molecular weight plasmid (14.17 kbp) with 0.73 MARI to two classes of antibiotics. Among the resistant bacilli, only 24.3% (MIC_90_ 500 mg/mL) and 8.1% (MBC_90_ 1000 mg/mL) were susceptible to honey with evidence of 14.85% and 5.94% significant viable reduction at 2 × MIC to less than 2.50 Log_10_ CFU/mL (*P* < 0.05). Only alkaloids significantly regressed (*P*=0.028) with susceptibility of resistant bacilli significantly correlate with bacteria inhibition (*r* = 0.534, *P*=0.049) at optimal cutoff limit of 0.32 mg/ml. Antibacterial honey with significant alkaloid biomarkers was detected at 3°10′0–3°30′0E and 6°30′0–7°30′0N of Southwest Nigeria. Spatial mapping evidently indicated variation in honey physicochemical and bioactive compounds and identified geographical locations suitable for production of anti-enteric honey rich in alkaloids marker required for prevention and treatment of resistant enteric bacilli infections.

## 1. Introduction

Use of honey as therapeutics in the last decades has received special attention due to it availability, affordability, and high efficacy against several infectious diseases [1]. Rapidly emerging antibiotic-resistant strains are fastly becoming a nightmare as they cause more morbidity and increased economic loss, particularly in intestinal infections [2]. Enteric infection is known as one of the leading major health challenges in many developing countries where sanitation standards remain poor [3]. There has been recorded success in treatment of enteric infections using antimicrobial agents. However, the rapid development of multidrug resistance is a concern with serious consequences on public health leading to increased treatment failures, as a result of antibiotic misuse and poor regulation [3]. The development of resistance is now inevitable due to acquisition of resistance-conferring DNA through highly mobile genetic elements (such as plasmids) [4]. High mobility of resistant plasmid DNA (R plasmid) enhances rapid dissemination of resistant strains with a related clonal trait through genetic exchange that intensifies infection burden in several communities [5].

In many Nigerian communities, the use of honey has become a popular local remedy for treating different intestinal and extraintestinal infections [6]. However, the antibacterial activities of few honey brands in Nigeria have been reported to depend mostly on the action of secondary metabolites (bioactive compounds) that include alkaloids, flavonoids, and phenol derivatives [7] and physicochemical parameters (such as pH, moisture, sugar composition, mineral contents, free acidity, water, total sugars, solids, and electrical conductivity) [8]. Despite the increase in use of honey for different therapeutics, adulteration of retail honey brands is a worried concern to many community residents. The geographical origin of some honey determines its acceptability and recommendation for bacterial infections. In addition, other factors such as processing methods, harvesting, storage conditions, the floral origin, plant species, environmental conditions, and floral diversity collectively prove to be major determinants for the quality of honey [8, 9].

A preliminary study had shown that some Nigeria honey possesses an effective antibacterial biomarker that could influence high inhibitory or cidal activities against resistant enteric bacilli [10] and enhance its identification for development of a novel antibacterial agent for intestinal infections. Not only quality and biomarker, but geospatial investigation of most suitable locations also favours high yield of honey with quality antibacterial biomarker which would enhance its large-scale production and development to combat the prevalent antibiotic-resistant bacterial strains [11]. Certain bioactive metabolites which are referred to as nonnutrient chemical compounds (phytochemicals) that could prevent microbial infections [12, 13] are needed to be evaluated for therapeutic application in severe enteric infections. In order to have reliable assessment of geographical locations that favour the production of anti-enteric honey, application of spatial mapping was utilized to enhance the identification of environmental factors, soil type, floral species, bee colonization, and favourable reproduction method. Therefore, geospatial distribution of Nigeria honey with reliable anti-enteric efficacy and potential antibacterial biomarkers as a novel candidate for development of a natural anti-enteric bacilli agent was studied.

## 2. Materials and Methods

### 2.1. Isolate Biotyping

Enteric bacilli (251 strains) obtained from the faecal sample of patients attending the out-patient department of the Federal Medical Centre, Abeokuta, were collected. This facility serves as a regional referral centre for internal medicine in Southwest Nigeria and also issued ethical approval for the study. Colonial and cell morphology of each strain was examined and further biotyped with an Analytical Profile Index for Enterobacteriaceae kit (API 20E).

### 2.2. Antibiogram

Strain susceptibility to the panel of commonly used antibiotics erythromycin (10 *μ*g), tetracycline (30 *μ*g), cefuroxime (30 *μ*g), augmentin (10/20 *μ*g), ceftazidime (30 *μ*g), gentamycin (10 *μ*g), ofloxacin (10 *μ*g), ampicillin (10 *μ*g), ciprofloxacin (10 *μ*g), and cotrimethaxazole (5/25 *μ*g) was determined using the Kirby–Bauer disc diffusion method [14]. The observed inhibition zones were interpreted, and the respective antibiotic minimum inhibitory concentrations (MICs) were determined in a standard broth microplate bioassay and were interpreted according to the CLSI guidelines [15].

### 2.3. R Plasmid Profile

Plasmid DNA from resistant strains was extracted using the alkaline lysis method [16] and separated by using completely submerged horizontal agarose gel electrophoresis at 60 mA and 220 V for 60 minutes. DNA bands were visualized with a UV transilluminator and profiled for respective molecular weights.

### 2.4. Antibiotic Resistance Relatedness

Level of resistance relatedness of the strains using multiantibiotic resistance index (MARI) values and R plasmid weight variation was evaluated by neighbor-joining dendrogram analysis constructed with the DendroUPGMA algorithm to produce a phylogram tree using the calculated similar distance matrix and coefficients to form different resistant cluster groups [17].

### 2.5. Honey Sampling

Different honey brands were collected from notable wholesale stores and markets patronized by large populace in Southwest Nigeria along with detail information traced to the production site was obtained, but adequate information on the type of bee producing the respective honey being retailed or demographic information of the bee-keepers could not be ascertained.

### 2.6. Physicochemical and Bioactive Compound Analysis

Only 23 collected unadulterated honey samples were evaluated for physicochemical parameters and phytochemical compounds using the spectrophotometric assay as previously described [18].

### 2.7. Antibacterial Activity and Time Kill Kinetics

Microtube dilution bioassay was used to determine the susceptibility of resistant biotypes to various honey samples. Briefly, to each 100 *μ*L serially diluted honey, 100 *μ*L of 0.5 MacFarland turbid resistant bacteria broth was added and then was incubated at 37°C in ambient air for 24 hours in a shaker set at 50 rpm. Absorbance of each well was measured at wavelength 590 nm before incubation and after incubation taking MIC at more than 95% inhibition after 24 hours while the MBC was also determined. The killing rate of the honey in 24 hours postinoculation was estimated in different honey dilutions at 6-hour postinoculation intervals for the estimation of total viable colony reduction [19, 20].

### 2.8. Geospatial Analysis

Production locations of each honey as described by the wholesalers and retailers were mapped according to coordinates (longitudes and latitudes) derived from the Goggle Earth satellite GIS. The average estimated values of bioactive compounds in each geographical location with their respective coordinates were aligned into the ArcGis programme for evaluation of the honey geographical distribution.

### 2.9. Data Analysis

Significance of resistance bacteria strains was determined by chi square (*χ*^*2*^) and descriptive analysis, and significance of physicochemical and bioactive metabolites were also determined using the *t*-test at *P* < 0.05. Bioactive and physicochemical parameters that induce inhibitory activity against resistant bacilli were predicted with multivariate regression analysis. Receiver operating characteristic (ROC) curve which operates on the coding of the strain as susceptible and resistance at optimal level of bioactive compounds and physicochemical activities causing inhibition was derived from the coordinates, and reliability at the area under the curve (AUC) drawn at values closer to 1 and unreliable at 0.50 or less was considered [21].

## 3. Results

### 3.1. Distribution of Enteric Bacilli and Antibiotic Susceptibility Profile

**Figure 1:**
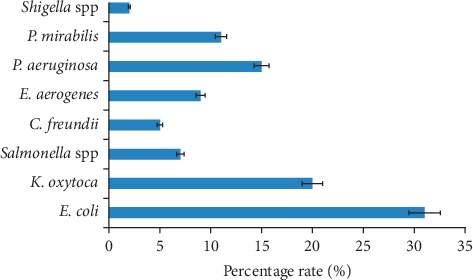
Distribution of enteric bacteria obtained from the subjects.

Only 62.6% showed significant resistance to cefuroxime, ampicillin (61.6%), and augmentin (54.2%) while more than 30% resistance at MIC ≥16 *μ*g/ml was to cotrimoxazole, ciprofloxacin, and tetracycline (*P*=0.004) (Figure 1 and Table 1).

### 3.2. Antibiotic Resistance Relatedness

**Figure 2:**
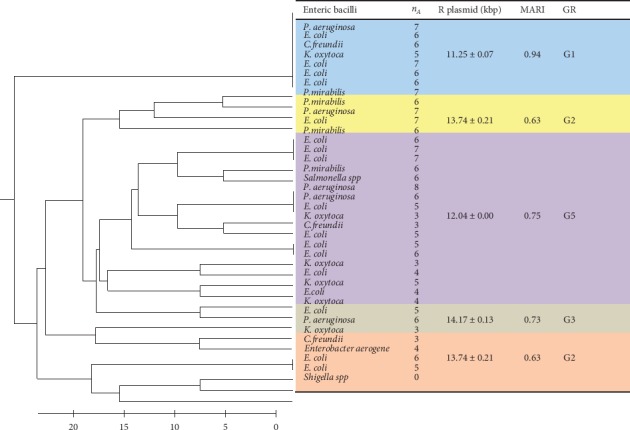
Resistance relatedness of enteric biotypes revealing different clusters of resistant phylogroups (GR, group of clustered resistant types; *n*_A_, number of resisted antibiotics; kbp, kilobase pair; MARI, multiantibiotic resistant index).

Resistant bacilli harbouring similar high R plasmid weight (13.74 kbp) and MARI of 0.63 clustered together into phylogroup 2 while group 3 clustered different high molecular weight plasmid (14.17 kbp) bacilli with MARI of 0.73. All the clustered groups showed resistance to more than two classes of antibiotics (Figure 2).

### 3.3. Antimicrobial Activities and Time Kill Rate

**Figure 3:**
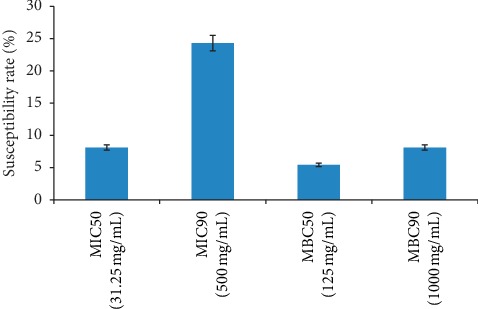
Susceptibility rate of resistant bacilli to Nigerian honey at various MIC and MBC levels.

**Figure 4:**
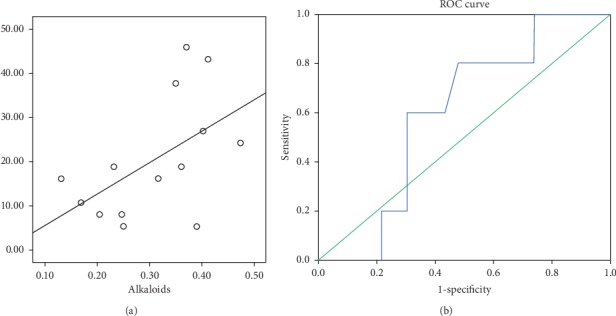
(a) Correlation coefficient of Nigerian honey antimicrobial activity against alkaloid content (*r* = 0.534, *P*=0.049). (b) Receiver operating characteristics to determine the optimal cutoff limit of alkaloid content in Nigeria honeys.

Significant overall anti-enteric susceptibility of 8.1% (MIC_50_ 31.25 mg/mL), 24.3% (MIC_90_ 500 mg/mL), 5.4% (MBC_50_ 500 mg/mL), and 8.1% (MBC_90_ 1000 mg/mL) (*P* < 0.05) was observed among the resistant strains as shown in Figure 3. Significant reduction in the average count of different bacilli to less than 3.00 Log_10_ CFU/mL was recorded at 2 × MIC after 24 hours of postinoculation compared to 1 × MIC and 1/2 × MIC (Table 2). Only alkaloids showed significant correlation (*r* = 0.534, *P*=0.049) and also regressed significantly (*P*=0.028) with bacteria inhibition with calculated cutoff limit of 0.32 mg/mL (Figure 4).

### 3.4. Geospatial Mapping

**Figure 5:**
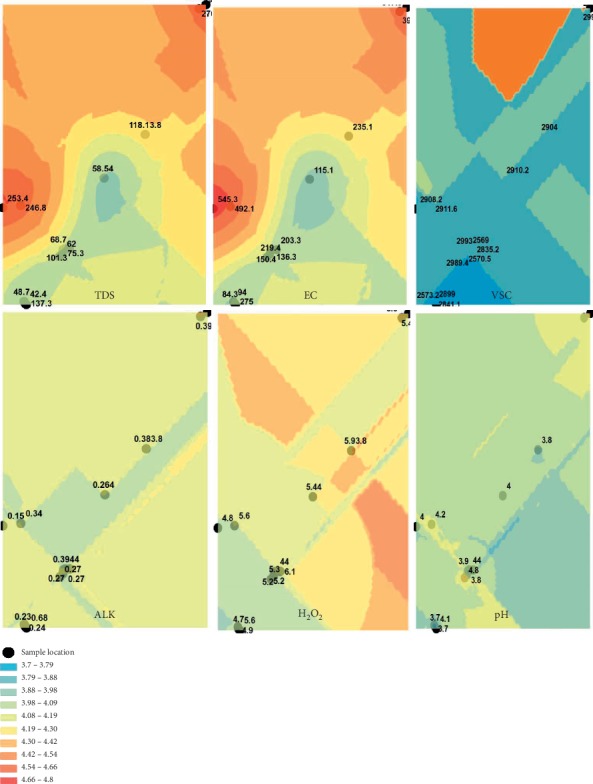
Spatial distribution of various honey sample parameters in Southwest Nigeria indicated by black spots (TDS, total dissolved solids; EC, electrical conductivity; VSC, viscosity; ALK, alkaloids; H_2_O_2_, hydrogen peroxide; pH).

Most honey samples collected within 3°50′0–4°30′0E and 7°50′0–8°50′0N geographical locations have similar viscosity range (2,910.42 to 2,929.13 cp), pH (3.7–3.79), hydrogen peroxide (5.70–5.82 mg/mL), and alkaloids (0.31–0.35 mg/mL) as mapped towards the southern part of the region as shown in Figure 5. Higher total dissolved solids were recorded among honey collected within 3°20′0–3°30′0E and 7°00′0–7°50′0N, and low electrical conductivity was also observed.

## 4. Discussion

Continuous spread of multiantibiotic-resistant *Escherichia coli*, *Klebsiella oxytoca*, and *Pseudomonas aeruginosa* which were recovered from the subjects indicate high prevalence of enteric bacilli pathotypes which could persistently increase enteric infections, mostly in children [22]. These pathotypes are propelling force for increasing enteric infectious diseases in many poor communities and is evidently considerable due to consumption of unhygenically prepared food and contaminated water. An observed resistance rate of more than 30% to commonly used antibiotics is a reflection of excessive and often unnecessary use of antibiotics caused by diverse multiple factors [23].

Neighbor-joining tree analysis evidently showed diverse resistant bacilli group harbouring similar high R plasmid weight and multiantibiotic resistance index (MARI), suggesting the spread of resistant enteric strains with the reservoir of resistant genetic material which can be readily transferred and spread among the populace [24]. This is identified as a driving factor that promotes rapid dissemination of resistance [1]. Not only is plasmid-mediated resistance a threat to public health, it also enhances persistent spread of community-acquired resistant bacilli [25] and emergence of new resistance pathotypes [26] characterised with high-level resistance diversity. Implication for R plasmid expression is the enhancement of gene recombination that could give rise to broad-scale spread, involving transposition and integration of other mobile genetic elements, thereby propelling high-level dissemination [27, 28].

Overall, significant MIC and MBC activity of all tested Nigerian honey is a considerable proof of anti-enteric bacterial activities of bioactive compounds with several synergistic mechanisms that could induce inhibition and cidal effect. Significant reduction in the average count to less than 3.00 Log_10_ CFU/mL at 2 × MIC against all the resistant bacilli after 24-hour postinoculation could conform the suitable dilution of honey that would effectively produce significant anti-enteric activity in infection related to gastrointestinal diseases. The evaluation of time kill kinetics further showed the growth inhibition rate and dose time-dependent mechanism [29]. Estimated low pH and high electrical conductivity provide the acidic component with very potent antibacterial activity [30], while low moisture content greatly contributed to the hygroscopic natures which enhance dehydration and death of bacteria cell [31]. A functional relationship of hydrogen peroxide to antibacterial activity is an important intrinsic factor needed because increased generation of hydroxyl ion radicals can cause DNA inhibition or degradation resulting in cytotoxic effects on bacteria cell metabolism [31]. Recorded significant variation in the level of antioxidants (flavonoids, alkaloids, terpenoids, and tannins) drives effective scavenging of reactive oxygen species (including superoxide anions and hydroxyl radicals), causing cellular toxicity and damage of functional organelles and inhibition of hydrogen bonding required for bacteria cell replication [32]. However, poor inference derived from antimicrobial intrinsic activity in honey in the recent past had steadily retrogressed its application for enteric infection. From the study, only alkaloids regressed and correlated significantly with susceptibility of resistant bacilli, which is indicated as the antibacterial marker for predicting Nigerian honey with anti-enteric activity. The bioactive compound of the alkaloid metabolite was reported to inhibit the mechanism of ATP-dependent efflux pumps activity [33] and actively form alkylated derivatives of ethyl- or methyl-pyrimidine and purine that could cause incorrect pairing during replication, resulting in mutation and possible inhibition of bacteria cell division and death [34]. Therefore, honey from Southwest Nigeria with significant alkaloids would serve as an effective antibacterial agent against resistant enteric bacilli.

Considering the geospatial mapping of physicochemical and bioactive parameters, it is evident that there is variability in several locations which also affects the composition and antimicrobial activity according to the type of flowering plant nectar, source of production, and environmental conditions [35]. Honey collected within 3°50′0–4°30′0E and 7°50′0–8°50′0N geographical locations has similar range of viscosity, pH, hydrogen peroxide, and alkaloid. This could have been caused by the floral type, abundance of bee species needed for nectar production, seasonal impact of humidity, pattern of rainfall, and temperature differences [36, 37].

Influence of climatic change, floral source, mineral contents, botanical origin, soil composition, environmental pollution, and extraction techniques amongst other factors usually dictates composition of phytochemical and physicochemical parameters in various honey [38]. More geographical information is still needed to verify the impact of ecological degradation, deforestation, seasonal honey bee reproduction, and plant types across notable rainforest locations in Southwest Nigeria [39] as identified by spatial mapping.

## 5. Conclusion

Spatial mapping evidently infers that this region is suitable for the production of antibacteria honey rich in alkaloids required for prevention and treatment of enteric infections caused by resistant bacilli. It would further enhance prevention and dissemination of multiantibiotic-resistance pathotypes in many Nigerian communities.

## Figures and Tables

**Table 1:** Antibiogram of enteric biotypes to commonly used antibiotics.

Antibiotics (*μ*g/mL)	Antibiotic susceptibility pattern (*N* = 406)				*χ* ^2^	*P* value
	S *n* (%)	I *n* (%)	R *n* (%)	MIC ^*∗*^(>16 *μ*g/ml) (*n*)		
Tetracycline	170 (41.9)	174 (42.9)	62 (15.3)	21 (33.9)		
Cefuroxime	62 (15.3)	90 (22.2)	254 (62.6)	35 (13.8)		
Augmentin	125 (30.8)	61 (15.0)	220 (54.2)	32 (14.6)		
Ceftazidime	181 (44.6)	110 (27.1)	115 (28.3)	29 (25.2)	31.094	0.004
Gentamycin	143 (35.2)	176 (43.3)	87 (21.4)	18 (20.7)		
Cotrimoxazole	165 (40.6)	147 (36.2)	94 (23.2)	42 (44.7)		
Ofloxacin	285 (70.2)	102 (25.1)	79 (19.5)	17 (21.5)		
Ampicillin	30 (7.4)	126 (31.0)	250 (61.6)	43 (17.2)		
Ciprofloxacin	188 (46.3)	128 (31.5)	90 (22.2)	35 (38.9)		

S = susceptible, I = intermediate, R = resistant, *N* = total number of enteric bacteria isolates obtained, *n* = number of bacteria isolates, and % = percentage of bacteria. ^*∗*^CLSI (2012). (*χ*^2^ = 31.094; *P*=0.009).

**Table 2:** Evaluation of time kill rate of Nigerian honeys.

Enteric isolates	Log_10_ CFU/mL								
	2 × MIC			1 × MIC			1/2 × MIC		
	0 h	12 h	24 h	0 h	12 h	24 h	0 h	12 h	24 h
*Escherichia coli*	5.01	3.32	2.50	5.02	3.38	2.79	5.70	3.52	3.12
*Salmonella species*	5.03	3.12	2.56	5.05	3.27	2.78	5.02	3.44	2.94
*Citrobacter freundii*	5.00	3.01	2.50	5.23	3.21	2.95	5.32	3.50	3.21
*Pseudomonas aeruginosa*	4.98	2.85	2.51	5.12	3.20	3.00	5.21	3.50	3.23
*Proteus mirabilis*	4.87	3.01	2.74	5.10	3.28	2.90	5.10	3.40	3.03
*Shigella species*	5.32	2.85	2.24	5.06	3.22	2.54	5.15	3.46	3.00

## Data Availability

The data used to support the findings of this study are available from the corresponding author upon request.
